# *NGF* and *P75^NTR^* Gene Expression Is Associated with the Hepatic Fibrosis Stage Due to Viral and Non-Viral Causes

**DOI:** 10.1371/journal.pone.0121754

**Published:** 2015-03-27

**Authors:** Ednelza da Silva Graça Amoras, Samara Tatielle Monteiro Gomes, Felipe Bonfim Freitas, Bárbara Brasil Santana, Geraldo Ishak, Marialva Tereza Ferreira de Araújo, Sâmia Demachki, Simone Regina Souza da Silva Conde, Marluísa de Oliveira Guimarães Ishak, Ricardo Ishak, Antonio Carlos Rosário Vallinoto

**Affiliations:** 1 Laboratory of Virology, Institute of Biological Sciences, Federal University of Pará (Universidade Federal do Pará—UFPA), Belém, Pará, Brazil; 2 João de Barros Barreto Hospital, Federal University of Pará (Universidade Federal do Pará—UFPA), Belém, Pará, Brazil; 3 School of Medicine, Institute of Health Sciences, Federal University of Pará (Universidade Federal do Pará—UFPA), Belém, Pará, Brazil; 4 Hepatology Outpatient Service, Holy House of Mercy Foundation of Pará (Santa Casa de Misericórdia do Pará), Belém, Pará, Brazil; University of Navarra School of Medicine and Center for Applied Medical Research (CIMA), SPAIN

## Abstract

This study evaluated the relative mRNA expression levels of nerve growth factor (NGF) and the p75 neurothrophin receptor (p75^NTR^) in different histological stages of human liver disease. Fifty-one liver biopsy specimens obtained from patients with hepatitis B virus (n = 6), hepatitis C virus (n = 28), and non-viral hepatitis – (n = 9) and standard histological liver (n = 8) as controls (CT) were subjected to qPCR and histopathological exams. Our data revealed a significant difference in the *NGF* expression levels between the three patient groups and the Control group. *p75^NTR^* expression levels in the HCV and NVH groups were higher than those observed in the HBV and Control groups. In cases of liver cirrhosis, higher *p75^NTR^* mRNA expression was observed, whereas NGF was expressed at higher levels in patients with hepatic fibrosis. *NGF* expression was lower in the F1 liver fibrosis stage, and p75^NTR^ receptor expression continuously and proportionately increased compared to the increase in the degree of fibrosis and was significantly higher in livers in fibrosis stages 3 and 4. The hepatic levels of NGF and p75^NTR^ were decreased and increased, respectively, relative to the stage of inflammatory activity. A positive correlation between p75^NTR^ and NGF gene expression was observed in livers with mild to moderate fibrosis, though not in cases of severe fibrosis and cirrhosis.

**Conclusion:**

Our results demonstrate that the course of chronic liver disease can be regulated by *NGF* and *p75^NTR^*, which function by decreasing or inhibiting hepatocyte regeneration and proliferation.

## Introduction

Viral hepatitis is one of the great pandemics of our time. Hepatitis B virus (HBV) and hepatitis C virus (HCV) are responsible for the vast majority of chronic liver diseases worldwide; therefore, they are important public health problems [1]. Approximately 170 million people, 3% of the world population, are carriers of chronic HCV infection, with different patterns of geographic distribution, while approximately 7% of the world population is chronically infected with HBV [1,2]. Due to persistent liver inflammation, the entire liver tissue undergoes a high rate of cellular destruction and regeneration, which results in an increased risk of developing conditions such as cirrhosis and hepatocellular carcinoma [3]. Liver injury triggered by HBV and HCV infection is primarily mediated by the host immune response to viral proteins expressed in infected hepatocytes and, to a lesser degree, the cytopathic effect directly caused by the virus [4].

As a consequence of chronic liver injury, quiescent hepatic stellate cells (HSCs), which store fat and vitamin A, undergo differentiation to an activated myofibroblastic phenotype under the action of pro-inflammatory cytokines, increasing synthesis levels of extracellular matrix components (i.e., collagens, elastin, proteoglycans and constituent proteins) [5,6]. This HSC activation is accompanied by a reorganization and expression of cytoskeletal proteins, such as α-smooth muscle actin (α-SMA), that acquire pro-fibrogenic properties [7]. The excessive deposition of extracellular matrix is the result of an imbalance between fibrogenesis and fibrolysis in the liver, and the proportion of deposited extracellular matrix becomes greater than the amount removed, especially collagen types I and II, proteoglycans, and glycoproteins [7].

Fibrosis is perceived as an initially beneficial physiological mechanism to limit the extent of the inflammatory process; however, if the fibrosis persists and becomes aggressive, it becomes pathological, leading to the distortion of the hepatic architecture [8]. Knowing the stage of liver fibrosis is essential for prognosis and to determine the appropriate antiviral therapy [9]. Patients without fibrosis or who manifest it at a minimum degree seem to progress slowly, and treatment could possibly be delayed or may be unnecessary. However, patients with significant degrees of fibrosis (septal or bridging) almost invariably progress to cirrhosis; in such cases, antiviral treatments should be strongly considered [10,11].

Nerve growth factor (NGF), discovered by Levi-Montalcini et al. in the 1950s, was originally characterized according to its capacity to stimulate the growth, differentiation, survival and maintenance of neurons during development and after injury [12,13]. NGF is a member of the neurotrophin (NT) family, which also includes brain-derived neurotrophic factor (BDNF), NT-3 and NT-4/5; NGF is the prototype and best-characterized member, both structurally and functionally [14]. The cellular response to NGF is determined by the combination of receptor expression: TrkA intermediates survival and differentiation, while p75 neurotrophin receptor (p75^NTR^), in the absence of TrkA, generally indicates the start of apoptosis [14]. Studies have shown that the components of the neurotrophin axis, NGF and p75^NTR^, are respectively expressed in hepatocytes and activated HSCs in normal and fibrotic liver in both humans and rats [15–19]. Studies have demonstrated that HSCs express p75^NTR^ and have undergone apoptosis in tissue culture in response to stimulation with recombinant NGF, suggesting that p75^NTR^ is a new marker of activated HSCs and that signaling by binding of this receptor may provide a selective apoptosis mechanism for HSCs [15–17].

Concerning the information already described about the involvement of NGF and P75NTR as immunological components which target apoptosis and inflammatory processes, we wondered if NGF and P75NTR expression could have some influence on liver tissue of HCV and HBV infected subjects. This study aimed to quantify *NGF* and *P75* ^*NTR*^ gene expression levels in liver biopsy specimens of patients with chronic hepatitis by HBV, HCV, and non-viral causes (NVH—non-viral hepatitis) to relate their possible roles with the clinical development of these infections and the various stages of the fibrosis process and hepatic inflammatory activity according to the METAVIR classification.

## Materials and Methods

### Study population

The study group consisted of 51 individuals, consecutive cases of chronic carriers of HBV (n = 6), HCV (n = 28) and NVH (n = 9) (including non-alcoholic liver disease, autoimmune hepatitis, and primary biliary cirrhosis, among others), seen at the Hepatology Outpatient Service of the Hospital of Holy House of Mercy Foundation of Pará (Fundação Santa Casa de Misericórdia do Pará—FSCMPA) and a Control group (n = 8) consisting of patients who underwent conventional biliary cholecystectomy without necro-inflammatory liver changes at the Surgery Service of João de Barros Barreto University Hospital (Hospital Universitário João de Barros Barreto) at the Federal University of Pará (Universidade Federal do Pará—UFPA).

All selected patients were clinically evaluated and were subjected to further investigation consisting of blood, biochemical, serological (HBsAg, HBeAg, anti-HBeAg, anti-HBctotal and anti-HCV), and virological (quantitative HCV-RNA and genotyping) tests, ultrasound and endoscopy, in addition to liver biopsy. These data were transcribed from clinical records to the study database. The criteria for inclusion of subjects in the study were 18 years of age or older, both genders, HBsAg carriers for more than 6 months, positive HCV-RNA carriers, with or without high values of alanine aminotransferase (ALT) and gamma-glutamyl transferase (GGT). Subjects who did not meet the aforementioned requirements and those patients co-infected with hepatitis D virus (HDV) and/or human immunodeficiency virus-1 (HIV-1) and patients who used or were using specific antiviral therapies against HBV or HCV were excluded from the research. This study was submitted and approved by the Research Ethics Committee of Holy House of Mercy Foundation of Pará, protocol Nos. 117/2009 and 684.432/2014, following the Guidelines and Rules for Research Involving Humans (Resolution 196 of the National Council of Health [Conselho Nacional de Saúde]). All subjects who agreed to participate in the study signed an Informed Consent Form (ICF).

### Obtained samples

Liver biopsy specimens were obtained from patients with medical indications for investigation of changes in the liver parenchyma. The biopsies were performed with a Tru-Cut needle and were guided by ultrasound. Each sample was divided into two parts, one of which was subjected to histopathological examination after hematoxylin-eosin (HE), chromotrope aniline blue (CAB), Gomori’s reticulin and Shikata’s orcein staining at the Department of Anatomic Pathology, UFPA. The diagnosis followed the classification of the Brazilian Society of Hepatology [20] and the French classification METAVIR [21], scoring the activity of the portal and periportal inflammatory infiltrates from 0 to 3 and any structural changes from 0 to 4. The other part of each biopsy specimen was sent for genetic study at the Laboratory of Virology/ICB/UFPA and was stored at −70°C until the time of use. Blood samples were also collected in vacuum tubes containing EDTA as an anticoagulant, and the plasma was separated by centrifugation and stored at −20°C until the time of use to assess ALT, aspartate aminotransferase (AST), GGT, alpha-fetoprotein and viral markers.

### RNA extraction

The fragment of liver tissue was kept in 500 μL of *RNA Later Tissue Collection* solution for RNA preservation and was subsequently extracted using a Norgen Biotek Corporation kit according to the manufacturer's recommended protocol. The sample purity was determined by the ratio between the absorbance measurements at 260 and 280 nm. A good extraction was considered in which the absorbance ratio ranged from 1.6 to 1.8. To observe the RNA purity and integrity, samples were loaded on 1% agarose gel in 1X TAE buffer stained with ethidium bromide (0.5 μg/mL) and subjected to electrophoresis at 80 V for 90 minutes. The observed bands correspond to the 28S, 18S and 5S rRNAs. The 28S and 18S major bands should appear on the gel at a 2:1 ratio. The 5S band should appear as faint as possible, indicating a low level of RNA degradation. To calculate the concentration, RNA was quantified by spectrophotometric reading on a Qubit 2.0 Fluorometer using Qubit RNA Assay Kits according to the manufacturer's protocol. For each sample, the concentration was adjusted to 50 ng/μL, and the sample was kept at −70°C until transcription.

### Reverse transcription (cDNA)

RNA samples were transcribed into complementary DNA (cDNA) using the *High-Capacity cDNA Reverse Transcription* kit (without inhibitor) (Life Technologies, Carlsbad, CA, USA). The cDNA reaction was prepared with a final volume of 20.0 μL containing 4.2 μL H_2_O, 2.0 μL buffer, 2.0 μL random primers, 0.8 μL dNTP Mix (100 mM), 1.0 μL reverse transcriptase (RT) enzyme (provided in the kit), and 10.0 μL extracted RNA. Subsequently, the mixture was placed in a Perkin-Elmer thermocycler machine (Cetus Corp., USA) and was submitted to cycling at 25°C for 10 minutes, 37°C for 120 minutes and 85°C for 5 minutes.

### mRNA quantification by Real-Time PCR (qPCR)

Real-time PCR (qPCR) was performed in 96-well plates using TaqMan reagents (Applied Biosystems, USA) in a Step One Plus machine (Life Technologies, Carlsbad, CA, USA). Expression assays for the *NGF* and *p75* ^*NTR*^ genes, with (glyceraldehyde-3-phosphate dehydrogenase) *GAPDH* as a reference gene, were performed in separate wells (singleplex) for both the patient and Control groups with primers obtained commercially from Life Technologies (Carlsbad, CA, USA). Each reaction consisted of 15 μL 2X TaqMan Universal PCR Master Mix, 1.5 μL 20X TaqMan Gene Expression Assay (NGF Hs00171458_m1 and p75^NTR^ Hs00609977_m1), 3 μL cDNA and 10.5 μL RNase-free water. The *GAPDH* gene (P/N 4326317E, Life Technologies, CA, USA) was used as a reference gene (endogenous control) to normalize the qPCR reactions.

The thermocycling conditions were 1 cycle of 2 min at 50°C followed by 10 min at 95°C, 40 cycles at 95°C for 15 sec and 60°C for 1 min. The relative expression level of each gene is presented as a multiple of the respective gene normalized to the Control sample.

In the standardization of the qPCR reactions, cDNAs and probes (endogenous and target genes) were titrated, aiming to calculate the amplification efficiencies of the reactions. For standardization, different cDNA concentrations were tested (neat and 4 dilutions of factor 2–1:2, 1:4, 1:8 and 1:16). All reactions were performed in triplicate. The same cDNA was simultaneously analyzed on plates (at different dilutions) with different probes to produce an efficiency curve to validate the 2^−ΔΔCT^ analysis method.

Relative quantification (RQ) of target genes and the calculation of the confidence interval were performed using the Comparative CT (ΔΔCT) method or the 2^−ΔΔ^ method, a method for RQ comparison of the exponential phase threshold without using the standard curve, where ΔΔCT = ΔCT_sample_- ΔCT_reference_ (Life Technologies, Foster City, CA, USA).

### Statistical procedures

All expression results are depicted in median values, and serum dosages are shown as averages. For statistical analysis, GraphPad Prism 5.0 [22] and BioEstat 5.0 [23] software programs were used. The differences between the groups were analyzed with the Kruskal-Wallis test and the Mann-Whitney U-test, as appropriate. Relationships between two variables were determined by Spearman correlation analysis. The significance level was established at 5% (p-value ≤ 0.05).

## Results

In the three patient groups and the Control group, the ALT and GGT levels, the stage of liver fibrosis, and inflammatory activity were assessed (Table 1). Mean ALT levels were higher in the groups of patients with HCV (89 IU/L) and NVH (97 IU/L); the same was observed for GGT (78 IU/L and 179 IU/L, respectively). Comparisons of ALT values revealed significant differences between HBV vs. HCV (p = 0.005), HBV vs. NVH (p = 0.012), HCV vs. NVH (p = 0.033), HCV vs. Control (p = 0.004) and NVH vs. Control (p = 0.0007) groups.

**Table 1:** Clinical and laboratory information study participants with and without HBV and HCV, with different degrees of disease.

Variables	HBV chronic hepatitis	HCV chronic hepatitis	Non-viral chronic hepatitis	Normal control
Study subjects (*n*)	6	28	9	8
Gender (F/M)	4/2	16/12	5/4	5/3
ALT (UI/L) Mean ± SD	35.8 ± 60.8	89 ± 97.1	97 ± 76.21	27 ± 8.11
Median	30.5	56	96	27
GGT (UI/L) Mean ± SD	30 ± 14.7	78 ± 98.8	170 ± 141.77	29 ± 10.74
Median	20.5	55	155	29
Fibrosis Stage^a^				
F0 (%)	1 (16.7)	2 (7.1)	1 (16.7)	8 (100.0)
F1 (%)	4 (66.6)	13 (46.5)	2 (33.3)	-
F2 (%)	-	7 (25.0)	-	-
F3 (%)	-	3 (10.7)	1 (16.7)	-
F4 (%)	1 (16.7)	3(10.7)	2 (33.3)	-
Inflammation Stage^b^				
A0 (%)	1 (16.7)	4 (14.3)	1 (16.7)	8 (100.0)
A1 (%)	4 (66.6)	13 (46.4)	4 (66.6)	-
A2 (%)	1 (16.7)	11 (39.3)	1 (16.7)	-

ALT: alanine aminotransferase; GGT: gamma-glutamyl transferase;

^a^Fibrosis Stages—F0: no fibrosis; F1: portal without septa; F2: portal with some septa; F3: many septa without cirrhosis; F4: cirrhosis;

^b^Inflammation Stages—A0: absent; A1: mild; A2: moderate.

Comparisons of GGT serum levels revealed significant differences among all groups (HBV vs. HCV [p = 0.016], HBV vs. NVH [p = 0.0003], HCV vs. NVH [p = 0.003], HCV vs. Control [p = 0.021] and NVH vs. Control [p = 0.0003]). Comparisons of ALT and GGT levels between HBV and Control groups were not significant (p > 0.05).

Among HCV patients, 53.6% were classified as F0 and F1 stages according to fibrosis level. Among patients with NVH, 50% were classified as F0 and F1 fibrosis stages and 50% were classified as stages F3 and F4, whereas among HBV patients, only 16.7% were classified in the F4 stage of fibrosis.

Evaluation of the degree of inflammatory response revealed that 83.3% of HBV and NVH patients were in the A1 and A2 stages, similar to what was observed among HCV patients (85.7%). In the Control group, 100% were classified as A0 stage (Table 1).

The *NGF* and *p75* ^*NTR*^ mRNA expression levels were measured, and the values are expressed as fold changes relative to the reference calibrator.

**Fig 1:**
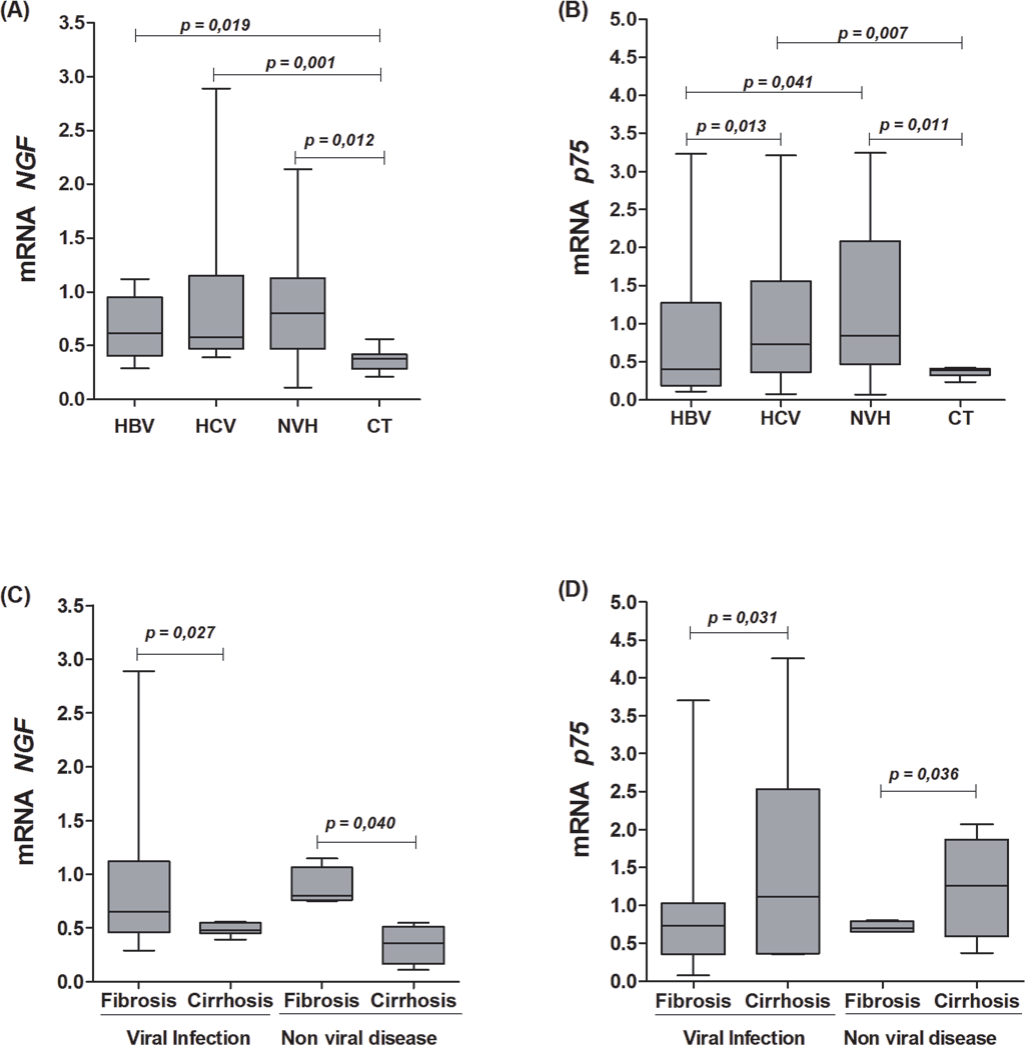
mRNA levels of *NGF* in the liver tissues. A: mRNA levels of *NGF* in the liver tissues of subgroups with HBV, HCV and NVH and the Control group (CT). B: mRNA levels of p75^*NTR*^ in the liver tissues of subgroups with HBV, HCV and NVH and the Control group (CT). C, D: mRNA levels of *NGF* and *p75* ^*NTR*^ between the groups with hepatic fibrosis and cirrhosis due to viral and non-viral causes.

Fig. 1A shows that *NGF* mRNA levels were significantly higher in patients with HBV, HCV, and NVH when compared to the Control group. The same pattern was observed for *p75* ^*NTR*^ mRNA expression (Fig. 1B). In the latter case, an increasing trend in mRNA expression was observed (in the ascending order of HBV < HCV < NVH); the differences between the HCV and NVH groups were statistically significant.

When mRNA levels were assessed according to the clinical conditions of liver fibrosis (without cirrhosis) or cirrhosis (Fig. 1C and 1D), the *NGF* mRNA levels were higher in the group with fibrosis than in the group with cirrhosis (p = 0.027 and p = 0040), whereas *p75* ^*NTR*^ expression levels were the opposite, with higher expression in patients with cirrhosis than in the group with liver fibrosis; these differences were significant (p = 0.031 and p = 0.036), regardless of viral or non-viral disease.

**Fig 2:**
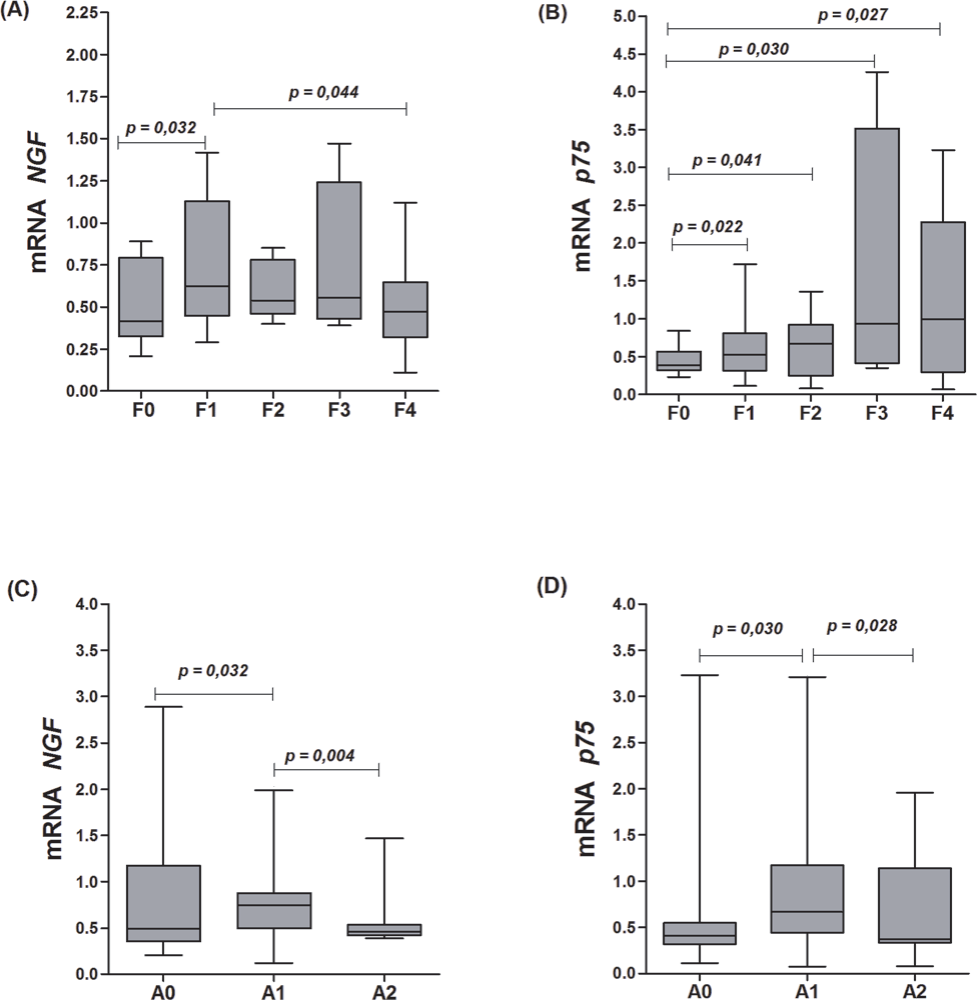
mRNA levels of *NGF* according to the clinical conditions of liver. A-D: mRNA levels of *NGF* and *p75* ^*NTR*^ according to the stage of fibrosis (F0 to F4) and inflammatory activity (A0 to A2) in the livers of all individuals with liver disease in the study population.

The grouping of all subjects (HBV, HCV and NVH) for the purpose of comparative analyses of fibrosis stage and hepatic inflammation and *NGF* and *p75* ^*NTR*^ expression levels is depicted in Fig. 2. Regarding the degree of fibrosis, *NGF* was most expressed in F1 to F3 fibrosis, with lower expression in F0 and F4 groups (Fig. 1A). *p75* ^*NTR*^ had lower mRNA expression levels in tissues without fibrosis (F0), with a sequential and a significant increase from F1 to F4 fibrosis stages (Fig. 2B).

According to the inflammatory process, *NGF* mRNA expression levels were higher in tissues at the A0 stage, and values decreased significantly to the A2 stage (Fig. 2C). Furthermore, *p75* ^*NTR*^ mRNA expression levels were higher in liver samples with A1 and A2 stage inflammatory activities and lower in liver samples without inflammation (stage A0) (Fig. 2D).

Significant associations were observed between high ALT levels (p = 0.041) and normal GGT levels (p = 0.028) with increased hepatic expression of NGF, while p75^NTR^ was expressed at higher levels (p = 0.012) in the livers of patients with high GGT levels (Table 2).

**Table 2:** Comparison of median values of relative *NGF* and *p75* ^*NTR*^ gene expression with serum levels of liver enzymes in individuals with and without HBV and HCV, with different degrees of the disease.

Gene	Normal ALT	High ALT	*p*	Normal GGT	High GGT	*p*
	(n = 26)	(n = 17)		(n = 19)	(n = 24)	
*NGF*	0.58	0.77	0.041^a^	0.80	0.55	0.028^b^
*p75* ^*NTR*^	0.82	0.56	0.55^c^	0.62	0.81	0.012^d^

ALT: alanine aminotransferase (normal: 14 to 55 UI/L); GGT: gamma-glutamyl transferase (normal: < 50 UI/L);

^a^NGF (normal *vs*. high ALT);

^b^NGF (normal *vs*. high GGT);

^c^ p75^NTR^ (normal *vs*. high ALT);

^d^ p75^NTR^ (normal *vs*. high GGT).

**Fig 3:**
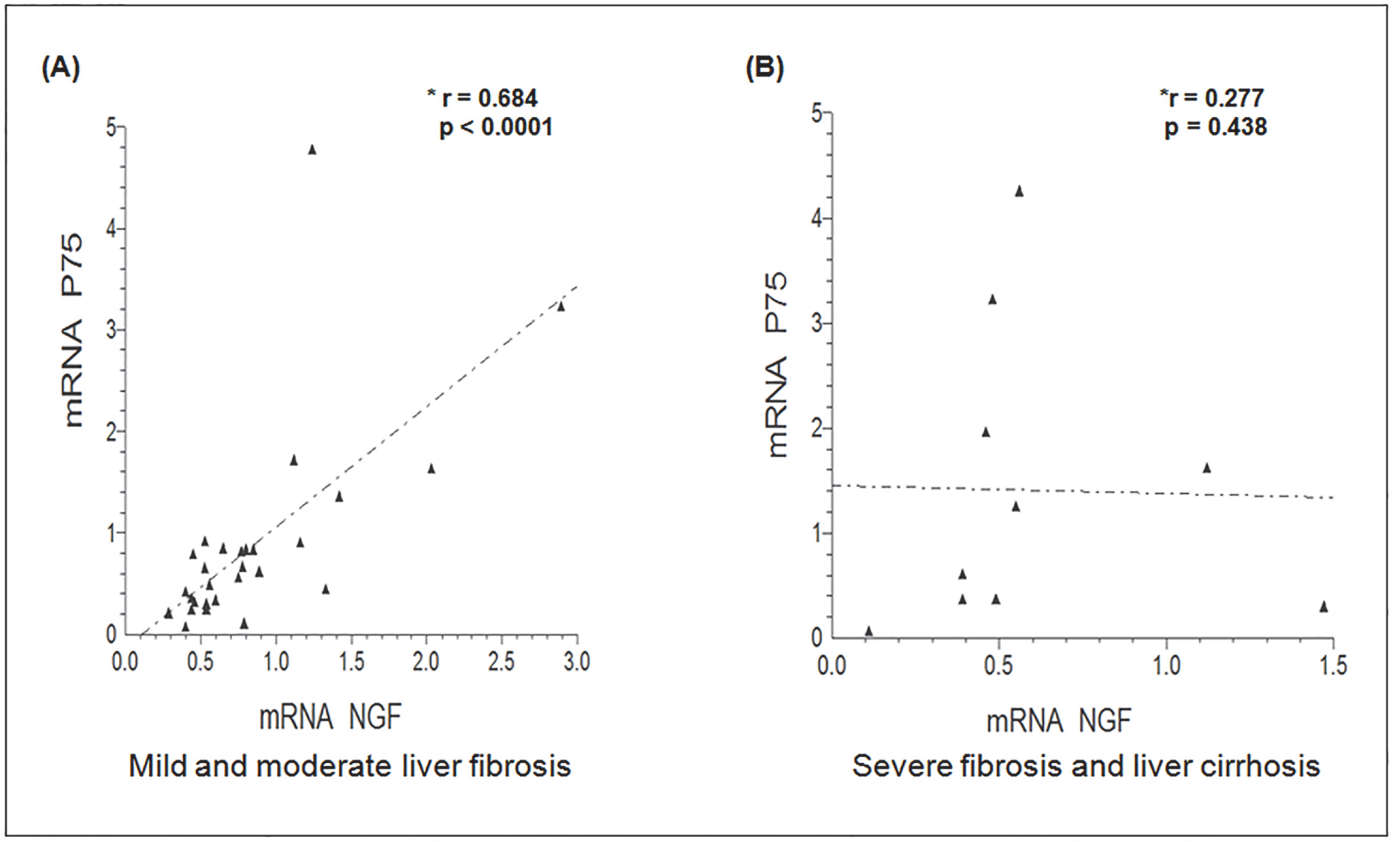
Correlation of *p75* ^*NTR*^ and *NGF* mRNA levels with fibrosis and cirrhosis stages. A, B: Spearman correlation analysis with mild to moderate fibrosis stages and severe fibrosis and cirrhosis stages.

A positive correlation was observed between *p75* ^*NTR*^ and *NGF* mRNA expression levels in liver tissue (Fig. 3A) with mild to moderate fibrosis (p <0.0001); however, this correlation was not observed in tissues with severe liver fibrosis and cirrhosis (Fig. 3B).

## Discussion

In this study, we found that the *NGF* and *p75* ^*NTR*^ mRNA expression levels in liver tissues in chronic HBV, HCV and NVH carriers were significantly higher when compared with normal liver biopsies. Our results partially disagree with *in vitro* studies and studies of experimentally induced liver fibrosis [16,17,20], which showed the expression of these genes only in fibrous liver tissue or after partial hepatectomy. Our results corroborate the findings of Trim et al. [15] and Kendall et al. [19], who used immunohistochemistry in normal liver to identify perisinusoidal cells with morphologies consistent with HSCs and p75^NTR^ positivity.

We observed that *NGF* mRNA expression was higher in subjects with hepatic fibrosis without cirrhosis compared to the group with cirrhosis, suggesting that *NGF* is expressed by hepatocytes in a state of regeneration and proliferation, while it is clear that *NGF* mRNA levels are lower in cirrhotic liver, where this regeneration process is reduced or absent [24,25]. In contrast to *NGF*, *p75* ^*NTR*^ mRNA expression was higher in subjects with cirrhosis compared to patients with fibrosis only, with a correlation between *p75* ^*NTR*^ expression and the reactivity of activated HSCs [26], similar to other *in vitro* experimental fibrosis studies [15–19].

As expected, there was an association between higher *NGF* mRNA expression levels and high ALT and normal GGT serum concentrations, while *p75* ^*NTR*^ was associated with high GGT concentrations. ALT is commonly high in chronic hepatocellular damage; however, with progression to fibrosis, ALT activity is typically reduced [27,28]. Although some patients with normal liver enzymes had severe fibrosis or even cirrhosis, most of them had a moderate liver disease caused by HCV.

A common feature of the study population was chronic liver injury with the absence of any prior drug therapy. Thus, we have evaluated the *NGF* and *p75* ^*NTR*^ mRNA expression levels in relation to the degree of fibrosis and hepatic inflammatory activity, showing associations between the expression levels of these two genes and the progression of liver injury. In the first stage of liver fibrosis and inflammation (F1/A1), *NGF* mRNA levels were significantly higher than in other liver injury stages, and *p75NTR* mRNA levels were lower. This result maybe is due to the fact that in the early stages of liver fibrosis, activation of human HSCs which is mediated by p75^NTR^ stimulates the release of growth factors such as hepatocyte growth factor (HGF), and stimulates hepatocyte regeneration and proliferation [25,29].

We observed a direct, progressive increase in *p75* ^*NTR*^ mRNA levels with the progression from fibrosis to cirrhosis, demonstrating that activated HSCs, mediated by p75^NTR^ expression, can mediate both the start and end of liver regeneration. In the early stages of liver regeneration, activated HSCs contain high HGF levels; this strongly mitogenic growth factor can substitute for the effects of transforming growth factor (TGF)-β1, the major hepatocyte antiproliferative factor produced by these cells [30]. In contrast, in the terminal stages of liver scarring, activated HSCs produce high TGF-β1 levels, triggering the start of an autocrine signaling cycle that perpetuates the activation of HSCs and becomes a barrier to liver regeneration [30].

Differing from our study, some results with experimental injuries induced by carbon tetrachloride (CCl_4_) and *in vitro* studies strongly suggest the pro-apoptotic role of NGF, directly regulating apoptosis of activated HSCs and mediated by p75^NTR^, which would represent a potential determining factor in liver fibrosis resolution. However, these results are best characterized only in self-limited injuries [16]. A positive correlation between p75^NTR^ and NGF expression was identified in the early stages of liver fibrosis, characterizing a likely paracrine action of activated HSCs mediated by p75^NTR^ in the compensatory regeneration of hepatocytes via NGF; however, this association was not sustained under severe liver fibrosis and cirrhosis conditions. In the livers of patients with cirrhosis caused by HCV, Novo et al. found that activated HSCs are resistant to most pro-apoptotic stimuli, including NGF neurotrophin due to overexpression of the Bcl-2 anti-apoptotic marker present in liver tissue; this characteristic may play a key role in the progression of fibrosis in chronic liver diseases [31].

Therefore, we suggest that, in accordance with the profile of the study population, our gene expression results could be also the product of other stimuli in livers damaged by viral persistence of HCV, HBV and other chronic infections because HSCs express receptors for HCV, such as CD80 and LDL (low-density lipoprotein) receptor, allowing an increase in the viral infection rate. The expression of non-structural proteins and core HCV proteins induces stellate cell proliferation and the release of inflammatory signals [32,33]. In nonalcoholic fatty liver disease, adipokines may mediate hepatic fibrogenesis [34,35]. As an example of these adipokines, leptin, a circulating adipogenic hormone, promotes fibrogenesis in HSCs [36–38]. Equally important for the development of liver fibrosis is insulin resistance, which is related to steatosis [39,40] and oxidative stress, is associated with ethanol metabolism, and is an important stimulus for hepatic fibrogenesis [41].

Taken as a whole, our results demonstrate that in the course of chronic liver disease in the study population, activated HSCs are negatively regulated by p75^NTR^-mediated inhibition of hepatocyte proliferation, which can be modulated by viral and non-viral components and supported by the persistence of aggression to the liver. The degree of histological fibrosis is an important marker of the disease stage [10,11], as the natural history of hepatitis B and C involves the gradual progression of hepatic fibrosis, which can eventually lead to cirrhosis, where most related complications occur [3]. In this context, the individual knowledge of p75^NTR^ expression levels would be a potent marker for the progression of hepatic fibrosis. However, we understand that it would be necessary to understand the gene expression mechanisms of this neurotrophin and its receptor subsequent to already established antiviral therapies. Finally, regarding the small number of subjects enrolled in the present study, specifically in the HBV group, which could not represent the molecular pathogenesis of the fibrotic transition to severe stages, we suggest that a follow up study should be conducted with a large number of patients, aiming to confirm our results.
